# The Depression, Anxiety, and Stress of Student-Athletes from a Pre- to Post-COVID-19 World

**DOI:** 10.3390/bs14080642

**Published:** 2024-07-25

**Authors:** Georgia A. Bird, Jennifer Cumming, Mary L. Quinton

**Affiliations:** School of Sport, Exercise, and Rehabilitation Sciences, University of Birmingham, Edgbaston, Birmingham B15 2TT, UK

**Keywords:** dual-career, depression, anxiety, stress, sport, confirmatory factor analysis

## Abstract

This study explored differences in student-athletes’ symptoms of depression, anxiety, and stress pre- to post-COVID-19-pandemic. The WHO reported a 25% increase in depression and anxiety rates worldwide, with young people disproportionately affected. Student-athletes face many stressors related to their sporting and academic feats, but what is not known is how the COVID-19 pandemic affected their experiences of symptoms of mental illness. A multiple-cohort cross-sectional study design was employed, and data collected using physical and online surveys. Participants (cohort 1 *M* age = 20.18 years, SD = 1.52; cohort 2 *M* age = 19.75 years, SD = 1.45) were recruited from UK universities (*N* = 807; 427 pre-pandemic cohort, 380 post-pandemic cohort). Results revealed statistically significant differences in mean depression (*F* (1, 805) = 23.92, *p* < 0.001), anxiety (*F* (1, 806) = 20.15, *p* < 0.001), and stress symptoms (*F* (1, 805) = 5.24, *p* = 0.022) scores between cohorts. Scores for the post-pandemic cohort were significantly higher than pre-pandemic, suggesting a worsening of symptom severity. Distributions of student-athletes across categories of symptom severity also worsened for depressive and anxiety symptoms post-pandemic and were skewed towards more severe categories. Symptoms of depression, anxiety, and stress were a concern pre-pandemic. Rates are higher in the post-pandemic cohort, suggesting a worsening of symptoms. These data add to evidence on student-athletes’ symptoms of mental illness by exploring a UK sample and comparing scores pre- and post-pandemic.

## 1. Introduction

The COVID-19 pandemic was a global issue that presented challenges for physical and mental health [1,2]. A national lockdown was announced on 23rd March 2020 in the UK whereby normal sporting activities were suspended as well as campus-based learning, presenting challenges for student-athletes alongside existing pressures [3]. Worldwide, symptoms of depression and anxiety rose by 25% during this period across all ages [4], and study-related stress was reported to increase in U.S. university students [5]. For the purpose of this study, depression is conceptualized as low positive affectivity and includes constructs such as dysphoria, hopelessness, and lack of interest. Anxiety relates to physiological hyperarousal, including autonomic arousal, situational anxiety, and anxious affect perceptions. Stress, on the other hand, refers to nervous arousal, difficulty to relax, and being easily upset and impatient [6,7]. Together, these constructs are an indicator of negative affectivity, reflecting the general levels of psychological distress experienced by a person [7].

Mental health problems due to the pandemic were also evidenced in multiple populations such as Italian students [8], those with pre-existing mental health disorders [9], and the Australian general population [10]. For students, it was widely assumed that university closures and ambiguity in exam procedures would impact their mental health [11]. It is unclear however, whether student-athletes followed this trend, given that young people were disproportionately affected by the pandemic [4,12]. It is, therefore, necessary for those working with athletes, including universities, to understand the impact of the pandemic on student-athlete mental health to ensure appropriate support is provided. Findings from this study will not only be beneficial for supporting student-athletes now but during potential future increased rates of COVID-19 lockdowns or periods of isolation (e.g., injury, outbreaks of other diseases). 

The national lockdown presented new and unfamiliar challenges for student-athletes alongside existing pressures of their dual-career identity [3]. Exasperating the situation further, student-athletes also experienced a loss of typical protective factors associated with their sport, such as training, social interaction, and learning, which was shown to contribute to anxiety about the future [13]. Despite an overall increase in symptom severity pre- to post-pandemic in North American samples [14], research suggests that athletes with greater protective factors may have been less severely impacted by the pandemic. For example, physical activity was found to be a protective factor for young people’s mental health and well-being during the pandemic [15]. Previous findings from North America also found that student-athletes with higher levels of social support and connectedness during the pandemic reported fewer problems with their mental health [16], and that student-athletes had somewhat better coping mechanisms than student non-athletes [14]. Given that the policies for COVID-19 restrictions differed across countries, there is a need to understand UK student-athletes’ symptoms of mental illness over COVID-19. 

It is rare that an opportunity arises to investigate what happens when key features of university and sport are removed, with the last instance of this situation relating to the suspension of all sports during World War II in the 1940s [17]. This knowledge could help to inform the provision of sports and mental health support in the future by exploring the historical macro-time component of the Process, Person, Context, Time (PPCT) model [18,19] to provide valuable insight into the effects this can have on student-athletes’ symptoms of mental illness. This endeavor would provide novel insight and extend current knowledge on how the ecological systems in which athletes exist influences their experience of symptoms of mental illness. Furthermore, as the time component (and particularly historical time) is often understudied, this study provides a theoretical contribution to the literature by expanding on sport psychology research, which has primarily focused on the context component of the model (e.g., [20]). 

Student-athletes must balance numerous and often competing demands that increase their risk for symptoms of mental illness [3]. However, there is mixed evidence in the literature, mainly drawn from North American samples, as to whether student-athletes are at less, as much, or greater risk for experiencing symptoms of mental illness than their non-athlete counterparts [21]. To broaden our understanding and better support UK student-athletes’ mental health, there is a need to explore prevalence rates within the context of the COVID-19 pandemic, as there is a gap for comparing rates of mental illness symptoms before and after the pandemic. Uncovering rates of mental illness symptoms post-pandemic is vital for ensuring that sufficient mental health support is provided to meet the needs of student-athletes.

Therefore, through a multiple-cohort cross-sectional design, the aim of this study is to investigate UK student-athletes’ symptoms of depression, anxiety, and stress pre- and post-COVID-19-pandemic. It was hypothesized that there would be higher symptoms of depression, anxiety, and stress in the post-pandemic cohort compared to the pre-pandemic cohort. 

## 2. Materials and Methods

### 2.1. Participants 

Following ethical approval for each cohort, data were collected from 427 student-athletes in cohort 1 and 380 in cohort 2, all enrolled at UK universities. For cohort 1, student-athletes were on average 20.18 years old (SD = 1.52), whereas those in cohort 2 were on average 19.75 years old (SD = 1.45). See Table 1 for a further breakdown of sociodemographic characteristics across the cohorts. 

### 2.2. Procedures and Data Collection 

Data were obtained from two separate cohorts as part of larger studies using a multiple-cohort cross-sectional study design. The pre-pandemic cohort completed questionnaires between January 2019 and March 2020 (*n* = 427). The post-pandemic cohort completed questionnaires between November 2021 and November 2022 (*n* = 380), shortly after restrictions were lifted in the UK for the last time. Research has found mixed evidence on the mental health implications at different points across academic years. For example, 1st-year university students have been found to experience increased symptoms of depression and anxiety over the academic year [22,23]. Conversely, elite student-athletes reported decreases in symptoms of anxiety and depression [24]. Consequently, including a broad data collection period in the present study enables us to capture differing experiences that may occur in the periods before and after the pandemic. 

### 2.3. Measures

#### 2.3.1. Sociodemographic Information

Participants provided information about their age, gender, sport type (i.e., individual vs. team), and competitive level (i.e., recreational, club, regional, or elite). 

#### 2.3.2. Symptoms of Depression, Anxiety, and Stress

Participants completed the shortened version of the Depression, Anxiety, and Stress Scale (DASS-21) [6], a 21-item measure with 7 items each for depressive (e.g., “I felt that I had nothing to look forward to”), anxiety (e.g., “I felt I was close to panic”), and stress (e.g., “I found it hard to wind down”) symptoms, reported on a Likert-type scale from 0 “did not apply to me at all” to 3 “applied to me very much or most of the time”. In addition to using subscale scores, the DASS-21 also allows for the simultaneous use of a total score (i.e., an indicator of general negative affectivity). 

The DASS-21 has been validated for use in athlete populations during and post-COVID-19 [25]. Using exploratory structural equation modeling (ESEM), a bifactor model (i.e., tapping general negative-affect and specific depression, anxiety, and stress factors simultaneously) provided the best fit for the data across two samples (sample 1 = 894; sample 2 = 589), and invariance testing suggested equivalence across gender, competitive level, sport type, and injury status. In addition to demonstrating acceptable internal reliability, the data showed convergent validity, with depression scores predicting mood states and all three subscales predicting sport-specific measures of anxiety, burnout, and psychological strain. The authors concluded that the DASS-21 is a psychometrically sound instrument for investigating the implications of COVID-19 in athletes. 

### 2.4. Statistical Analysis 

Using SPSS v.29, data were cleaned and screened for missing data and outliers [26]. Item-level descriptives statistics were calculated (i.e., mean, standard deviation, skewness, and kurtosis) for each cohort. Internal reliability was calculated using Cronbach’s alpha coefficients separately for each cohort, with values over 0.70 indicating acceptable levels of internal consistency [27]. Confirmatory factor analysis (CFA) was used to confirm the bifactor model of both samples using AMOS 29.0 data analysis software with maximum likelihood estimate. To account for multivariate non-normality, determined by Mardia’s normalised kurtosis coefficient (critical ratio > 1.96), parametric bootstrapping of 2000 samples was used for parameter estimation. The model’s overall goodness of fit was tested using the chi-squared (χ^2^) likelihood ratio statistic (*p* > 0.05). Because a nonsignificant χ^2^ is rarely obtained in practice, additional fit indices were reported [28]. Model fit was therefore also determined by the combination of normed chi-square statistics (χ^2^/df < 3), root mean square residual (RMSEA ≤ 0.08), comparative fit index (CFI ≥ 0.90), Tucker–Lewis index (TLI ≥ 0.90), and normed fit index (NFI ≥ 0.90) [28]. Standardised factor loadings for items loading on its intended subscale and the general negative affectivity scale are reported along with correlations between the subscales. 

For the main analyses, depression, anxiety, and stress were continuous variables and used to conduct multivariate analysis of variance (MANOVA) tests for comparing mean scores across cohorts and sociodemographic variables, with scores multiplied by 2 in line with authors’ recommendations [6]. ANOVAs were also conducted to compare these differences by the general negative affectivity score, with all items summed. The subscales were also transformed into categorical variables to understand the distribution of student-athletes across symptom severity from normal through mild, moderate, severe, and extremely severe symptoms (Table 2) [6]. Cross-tabulations and chi-squared tests were conducted on the categorical variables to assess for statistically significant differences in symptom severity pre- to post-pandemic. Pearson correlations were conducted to assess the relationship between age and mental illness symptoms. For all relevant analyses, the probability level was set at *p* = 0.05 and 95% confidence intervals (CI) were reported. 

## 3. Results

### 3.1. Preliminary Analyses and Internal Reliability 

DASS-21 items ranged between 0.23 and 1.34 (cohort 1) and 0.51 and 1.33 (cohort 2). Standard deviations of all items across both cohorts were less than 1.0. All items fell within acceptable levels of tolerance for skewness (i.e., between −3 and +3) and kurtosis (−10 to +10) when using structural equation modelling (SEM) [29]. Item descriptives are reported in Supplementary Table S1. 

Mardia’s coefficient (cohort 1 = 124.55, c.r. = 41.40; cohort = 112.68, c.r. = 34.34) indicated significant departures from multivariate normality [30]. Bootstrapping of 2000 samples was therefore used in both cohorts for parameter estimation. 

The internal reliability, as measured by Cronbach alpha coefficients, was acceptable (α > 0.70) for both cohorts: depression (cohort 1 = 0.85, cohort 2 = 0.91), anxiety (cohort 1 = 0.74, cohort 2 = 0.83), stress (cohort 1 = 0.82, cohort 2 = 0.86), and negative affectivity (cohort 1 = 0.90, cohort 2 = 0.94). A summary of the internal reliability information, including the corrected item-total correlations, Cronbach’s alpha if item deleted, and Cronbach’s alpha with 95% confidence intervals, can be found in Supplementary Table S2. 

### 3.2. Factor Structure 

Table 3 shows the model fit values for the DASS-21 bifactor model tested. For both cohorts, χ^2^/df, RMSEA, CFI, TLI, and NFI indicated good fit for the bifactor model. The standardised factor loadings ranged from poor to excellent (i.e., −0.20 to 0.72 for cohort 1 and −0.23 to 0.84 for cohort 2). Aside from the depression subscale in cohort 1, standardised factor loadings were generally higher on the total/general negative affectivity scale than for the specific factor. Correlations between the latent factors ranged from −0.124 to 0.448 (cohort 1) and 0.484 to 0.603 (cohort 2) (see Supplementary Table S3).

### 3.3. Cohort Differences in Depression, Anxiety, Stress, and General Negative Affectivity 

Pre-pandemic mean total scores for symptoms of mental illness reflected normal symptoms of stress (*M* = 12.94, SD = 7.87) and depression (*M* = 7.63, SD = 6.97), and normal/mild symptoms of anxiety (*M* = 7.75, SD = 6.72). Post-pandemic, these mean total scores were normal/mild for stress (*M* = 14.32, SD = 9.22), mild for depressive symptoms (*M* = 10.51, SD = 9.69), and moderate for anxiety symptoms (*M* = 10.17, SD = 8.58).

**Figure 1:**
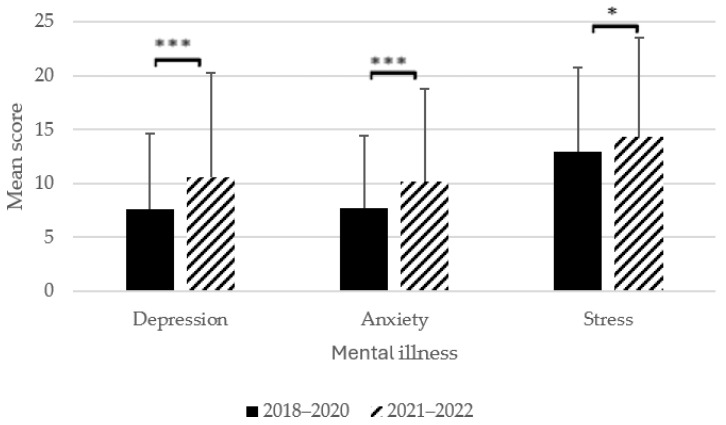
Changes in mean depression, anxiety, and stress scores from pre- to post-pandemic. Note, * *p* < 0.05, *** *p* < 0.001. Range of total mean scores was 0–42 for stress, 0–38 for anxiety, and 0–42 for depression symptoms.

Results of the MANOVA revealed that student-athletes post-pandemic reported higher levels of mental illness symptoms compared to the pre-pandemic cohort (Pillai’s trace = 0.040, *F* (3, 803) = 11.28, *p* < 0.001, *Ƞ*^2^_p_ = 0.040, observed power = 99.9%). At the univariate level, these differences were statistically significant for depressive (*F* (1, 805) = 23.92, *p* < 0.001, *Ƞ*^2^_p_ = 0.029, observed power = 99.8%), anxiety (*F* (1, 806) = 20.15, *p* < 0.001, *Ƞ*^2^_p_ = 0.024, observed power = 99.4%), and stress symptoms (*F* (1, 805) = 5.24, *p* = 0.022, *Ƞ*^2^_p_ = 0.006, observed power = 62.8%) (Figure 1). That is, student-athletes reported higher symptoms of depression, anxiety, and stress in the post-pandemic cohort.

For general negative affectivity, there was also a significant cohort difference (*F* (1, 806) = 18.75, *p* < 0.001, *Ƞ*^2^_p_ = 0.023, 95% CI (0.01, 0.05)), where the post-pandemic cohort had higher scores (*M* = 17.50, SD = 12.54) than the pre-pandemic cohort (*M* = 14.16, SD = 9.30).

Cross-tabulations and chi-squared tests revealed statistically significant differences in the distribution of student-athletes across symptom severity (Table 4) for depression (χ^2^ = 29.28, df = 4, *p* < 0.001) and anxiety (χ^2^ = 27.86, df = 4, *p* < 0.001) from pre- to post-pandemic. There were no statistically significant differences in distribution for stress (χ^2^ = 9.45, df = 4, *p* = 0.051). Results highlight that student-athletes in the post-pandemic cohort reported proportionately greater symptoms of depression and anxiety compared to those at pre-pandemic. 

### 3.4. Differences and Relationships in Symptom Severity by Sociodemographic Variables

#### 3.4.1. Correlations with Age

For cohort 1, there were no significant relationships between age and symptoms of depression (*r* = 0.038, *p* = 0.437), anxiety (*r* = −0.079, *p* = 0.102), stress (*r* = 0.077, *p* = 0.113), or general negative affectivity (*r* = 0.018, *p* = 711). For cohort 2, there were no significant relationships between age and symptoms of anxiety (*r* = −0.081, *p* = 0.118), stress (*r* = −0.044, *p* = 0.396), or general negative affectivity (*r* = −0.084, *p* = 0.104). However, there was a significant correlation for depressive symptoms (*r* = −0.104, *p* = 0.044), indicating a relationship between younger student-athletes and greater symptoms of depression.

#### 3.4.2. MANOVAs (for DASS Subscales) and ANOVAs (for General Negative Affectivity) for Sociodemographic Variables of Cohort 1

Gender. There was a significant multivariate effect for gender (Pillai’s Trace = 0.051, *F* (3, 423) = 7.54, *p* < 0.001, *Ƞ*^2^_p_ = 0.051, observed power = 99%). At the univariate level, there were significant differences for symptoms of stress (*F* (1, 427) = 7.54, *p* = 0.006, *Ƞ*^2^_p_ = 0.017, observed power = 78%) and anxiety (*F* (1, 427) = 5.28, *p* = 0.022, *Ƞ*^2^_p_ = 0.012, observed power = 63%), but not depression (*F* (1, 427) = 1.00, *p* = 0.321, *Ƞ*^2^_p_ = 0.002, observed power = 17%). Females reported greater stress (*M* = 13.86, SD = 7.95) and anxiety symptoms (*M* = 8.41, SD = 7.33) compared to males (*M* = 11.77, SD = 7.62; *M* = 6.91, SD = 5.77, respectively). For general negative affectivity, there were no significant gender differences (*F* (1, 426) = 2.59, *p* = 0.108, *Ƞ*^2^_p_ = 0.006, 95% CI (0.00, 0.03)). 

Sport type. There was no significant multivariate effect for sport type (Pillai’s Trace = 0.002, *F* (3, 423) = 0.30, *p* = 0.827, *Ƞ*^2^_p_ = 0.002, observed power = 11%). For general negative affectivity, there were no significant sport type differences (*F* (1, 426) = 0.05, *p* = 0.820, *Ƞ*^2^_p_ = 0.001, 95% CI (0.00, 0.01)).

Competitive level. There was no significant multivariate effect for competitive level (Pillai’s Trace = 0.029, *F* (9, 1266) = 1.39, *p* = 0.186, *Ƞ*^2^_p_ = 0.010, observed power = 68%). For general negative affectivity, there were no significant competitive level differences (*F* (3, 425) = 1.17, *p* = 0.320, *Ƞ*^2^_p_ = 0.008, 95% CI (0.00, 0.03)).

#### 3.4.3. MANOVAs and ANOVAs for Sociodemographic Variables of Cohort 2

Gender. There was a significant multivariate effect for gender (Pillai’s Trace = 0.023, *F* (3, 375) = 2.96, *p* = 0.032, *Ƞ*^2^_p_ = 0.023, observed power = 70%). At the univariate level, there were significant differences for symptoms of stress (*F* (1, 379) = 6.20 *p* = 0.013, *Ƞ*^2^_p_ = 0.016, observed power = 70%) and anxiety (*F* (1, 379) = 4.60, *p* = 0.033, *Ƞ*^2^_p_ = 0.012, observed power = 57%), but not depression (*F* (1, 379) = 0.95, *p* = 0.331, *Ƞ*^2^_p_ = 0.003, observed power = 16%). Females reported greater stress (*M* = 15.08, SD = 9.40) and anxiety symptoms (*M* = 10.76, SD = 8.85) compared to males (*M* = 12.55, SD = 8.61; *M* = 8.73, SD = 7.71, respectively).

For general negative affectivity, there was a significant gender difference (*F* (1, 378) = 4.10, *p* = 0.044, *Ƞ*^2^_p_ = 0.011, 95% CI (0.00, 0.04)), where females had higher scores (*M* = 18.32, SD = 12.74) than males (*M* = 15.51, SD = 11.86).

Sport type. There was a significant multivariate effect for sport type (Pillai’s Trace = 0.022, *F* (3, 373) = 2.84, *p* = 0.038, *Ƞ*^2^_p_ = 0.022, observed power = 68%). At the univariate level, there were significant differences for symptoms of stress (*F* (1, 377) = 4.78 *p* = 0.029, *Ƞ*^2^_p_ = 0.013, observed power = 59%) and anxiety (*F* (1, 377) = 6.61, *p* = 0.011, *Ƞ*^2^_p_ = 0.017, observed power = 73%), but not depression (*F* (1, 377) = 1.45, *p* = 0.229, *Ƞ*^2^_p_ = 0.004, observed power = 23%). Individual athletes reported greater stress (*M* = 15.39, SD = 9.20) and anxiety symptoms (*M* = 11.35, SD = 9.14) compared to team athletes (*M* = 13.32, SD = 9.16; *M* = 9.09, SD = 7.86, respectively).

For general negative affectivity, there was a significant sport type difference (*F* (1, 376) = 4.61, *p* = 0.032, *Ƞ*^2^_p_ = 0.012, 95% CI (0.00, 0.04)), where individual athletes had higher scores (*M* = 18.93, SD = 13.13) than team athletes (*M* = 16.16, SD = 11.83).

Competitive level. There was no significant multivariate effect for competitive level (Pillai’s Trace = 0.026, *F* (9, 1113) = 1.09, *p* = 0.369, *Ƞ*^2^_p_ = 0.009, observed power = 55%).

For general negative affectivity, there were no significant competitive level differences (*F* (3, 374) = 1.87, *p* = 0.134, *Ƞ*^2^_p_ = 0.015, 95% CI (0.00, 0.04)).

## 4. Discussion

Previous research, predominantly from North American samples, indicate mixed results on the extent to which student-athletes are at risk for symptoms of mental illness [21]. As a contribution to the literature, the results of the present study clearly indicate that UK student-athletes are experiencing some difficulties with symptoms of depression, anxiety, and stress (mild to extremely severe) pre- but especially post-pandemic, and that there is an urgent need to support their mental health. Research has shown the impact of the COVID-19 pandemic on the mental health of North American student-athletes [14,16] and of UK 13–19 year olds [15]. Although the authors argue that athletes appeared to face fewer mental health challenges when compared to non-athletes, they did note that post-pandemic scores for anxiety were nonetheless higher than pre-pandemic values [14]. The present findings follow this trend of heightened post-pandemic symptoms and enhance our understanding of student-athlete mental health by providing novel knowledge on UK student-athletes’ symptoms of mental illness as a population who experience different health, educational, and sporting contexts to those in North America [21]. The findings are also situated within the time component of the PPCT model [18,19] and provide valuable insight into the effects this can have on student-athletes’ symptoms of mental illness. 

### 4.1. Rates of Depression, Anxiety, Stress, and Negative Affectivity 

Although COVID-19 was a major global stressor, on a smaller scale, student-athletes may experience other periods of isolation that mimic some of the features of the pandemic, such as the removal of sport protective factors. Symptoms of depression and anxiety were elevated over the COVID-19 pandemic, one of the many factors that has worsened mental health. In particular, depressive symptoms were heightened from normal to mild levels, anxiety from normal to mild, and stress from normal to borderline mild. Although severity of symptoms of stress are not statistically significantly different post-pandemic, the mean score has significantly increased. A key clinical implication of the present findings is that these symptoms will become worse without intervention, and that students may leave university with poor mental health that could have been supported, or they may drop out of university altogether [31]. To identify potential intervention targets, future research should explore protective factors for student-athletes for any future eventualities of pandemics or other stressors which relate to periods of isolation from sport (e.g., injury). 

For depressive and anxiety symptoms, the severity has significantly skewed towards more severe categories of symptoms from pre- to post-pandemic. Of student-athletes, 46.6% reported mild to extremely severe symptoms of depression post-pandemic compared to 32.8% pre-pandemic, and 52.6% reported mild to extremely severe symptoms of anxiety post-pandemic compared to 44.3% pre-pandemic. Alarmingly, rates of extremely severe symptoms of anxiety increased from 8% to 19.5%. Importantly, the mean score (*M* = 10.21) reflects mild levels of symptoms. If this alone was considered, then the proportion experiencing extremely severe symptoms would have been missed. There is emerging evidence that taking a more person-centered approach to research and looking at person-level changes may be better for supporting mental health [32]. For example, female student-athletes’ mental well-being has been found to fluctuate over time when investigated at the person level but not at the aggregate and group level [33]. Furthermore, and in line with the PPCT model, student-athletes have different experiences that may influence their mental health outcomes, and it is important to consider the individual and not generalize all student-athletes into normal or mild categories when 13.5% to 28.4% are experiencing those severe to extremely severe symptoms. These distinctions have important applied implications for supporting student-athletes’ mental health, because the intervention for someone with mild symptoms might be different from that for those experiencing more severe symptoms (i.e., campus counselling vs. clinical treatments [34,35]. 

Another reason explaining the worsening in symptoms between the cohorts could be the sociodemographic composition of the samples. It is well evidenced that certain individual characteristics, such as identifying as female and being an individual-sport athlete, can be risk factors towards poor mental health [36,37]. In the present study, cohort 1 contained 56% females and 37% individual-sport athletes, compared to 69% and 49% in cohort 2, respectively. Notably, females’ and individual athletes’ mean anxiety and stress scores in cohort 2 were greater than in cohort 1, and the higher proportion of females and individual athletes could have driven these findings. Collectively, aligned with the PPCT model, these findings support the importance of understanding how an athlete’s person factors (i.e., characteristics) might interact with the context (i.e., university as a student-athlete) and time (post-COVID-19-pandemic) to influence their mental health. Future qualitative research in this area would facilitate a more in-depth exploration of the nuances of these interactions to further extend a bioecological systems approach to mental health in sport and exercise psychology.

### 4.2. Psychometric Properties of the DASS-21

CFA revealed further support for the bifactor structure of the DASS-21 with non-clinical athlete populations [25]. Data from both cohorts show acceptable levels of fit by using a combination of indices. But similar to Vaughan et al. [25], we noted that many of the items had higher factor loadings on negative affectivity than their intended factors. This adds to growing arguments that the items are “not pure measures of each factor” (p. 10 [25] but also share unavoidable overlap with a shared conceptual core [7]. Indeed, Lovibond and Lovibond [6] explained that the DASS-21 captures core aspects of depression (e.g., anhedonia, dysphoria, hopelessness, and inertia), anxiety (e.g., autonomic arousal, skeletal musculature effects, situational anxiety, and subjective experiences of anxious affect), and stress (e.g., difficulty relaxing, nervous arousal, easily upset/agitated, and irritable/over-reacting) symptomology that will result in factor overlap. Based on these analyses, we agree that the DASS-21 is best conceptualized as a general negative affectivity scale that co-exists alongside depression, anxiety, and stress as separate factors. Further, acceptable levels of internal reliability via Cronbach’s alpha were found for the sub-scales and when all items were combined into an overall scale of negative affectivity. These findings contribute to the growing evidence that the DASS-21 is suitable for use with athletes and for exploring the impact of the COVID-19 pandemic within this population [25]. 

### 4.3. Strengths, Limitations, and Future Research

A strength of this study is its contribution to knowledge on UK student-athletes’ symptoms of mental illness, but particularly when faced with challenges presented by factors such as the COVID-19 pandemic. A key limitation of the present study is that it involved using a self-report measure within a cross-sectional design. That is, different cohorts participated at the two time points, providing only an indicator of recent mental illness symptomology (i.e., how the athletes felt over the last 2 weeks). It is possible that symptoms varied over time, and participants may have not wished to disclose symptoms for socially desirable reasons (e.g., to avoid stigma) [25,38].

Nevertheless, the heterogeneity of the sample provides opportunity to generalise the findings to student-athletes. Future research should also explore ways in which student-athlete mental health can be promoted, such as by exploring their symptoms of well-being and various risk and protective factors that are associated with their symptoms of mental illness. Recently, the DASS-21 has been shown to be a dependable and usable tool for monitoring progress in clinical settings, from both the perspectives of youth and clinicians [39]. Aligned with calls for an early-intervention framework for preventing and responding to athletes’ mental health needs [20], future research may similarly wish to examine the potential utility and dependability of using the DASS-21 as a screening and progress-monitoring tool in sport. As popularity grows for using the DASS-21 with student-athletes and athlete populations more widely, a systematic review to synthesise this research and draw conclusions around its applicability would also be a worthwhile endeavour.

### 4.4. Conclusions

Taken together, the results indicate that large proportions of student-athletes reported heightened internalising symptoms associated with depression, anxiety, and stress pre-pandemic, suggesting that they were struggling with their mental health. However, post-pandemic, the severity of symptoms was heightened for some student-athletes’ experiences of depression and anxiety. This clear increase in rates of student-athletes’ symptoms of mental illness alongside research showing its association with negative outcomes (e.g., functional impairment, social skill deficits, substance abuse [40]) warrants immediate attention to understand how best to support their mental health, considering the broader ecological system. 

Future research should aim to address this problem to understand how athletes, coaches, universities, and others who work with them can be aware of their symptoms of mental illness and support these athletes, but also to investigate whether interventions can be implemented to reduce the risk of such symptoms in the first instance during this peak developmental period. The findings highlight that severe implications can occur when key features of the sport environment are removed. Therefore, key, unique features of the sport environment should be researched to understand how they influence student-athletes’ mental health (symptoms of mental illness and well-being). 

## Figures and Tables

**Table 1:** Sociodemographic breakdown of study variables (and standard deviations).

Demographics	Depression	Anxiety	Stress	Negative Affectivity
	Cohort 1 (N = 427)			
Gender				
Male (*n* = 188)	8.00 (7.09)	6.91 (5.77)	11.77 (7.62)	13.34 (8.78)
Female (*n* = 239)	7.33 (6.87)	8.41 * (7.33) ^a^	13.86 ** (7.95) ^a^	14.80 (9.66)
Sport type				
Individual (*n* = 156)	7.84 (6.67)	7.90 (7.13)	12.85 (8.00)	14.29 (9.59)
Team (*n* = 271)	7.50 (7.14)	7.66 (6.49)	13.00 (7.80)	14.08 (9.15)
Competitive level				
Recreational (*n* = 53)	8.00 (6.63)	7.88 (6.70)	13.33 (7.95)	14.60 (9.25)
Club (*n* = 131)	7.12 (6.51)	7.48 (6.62)	12.50 (7.93)	13.55 (9.17)
Regional (*n* = 209)	7.40 (7.08)	7.91 (6.84)	12.70 (7.84)	14.01 (9.34)
Elite (*n* = 33)	10.48 (8.12)	7.66 (6.77)	15.58 (7.61)	16.86 (9.74)
	Cohort 2 (N = 380)			
Gender				
Male (*n* = 117)	9.75 (10.05)	8.73 (7.71)	12.55 (8.61)	15.51 (11.86)
Female (*n* = 262)	10.80 (9.52)	10.76 * (8.85) ^a^	15.08 * (9.40) ^a^	18.32 * (12.74) ^a^
Sport type				
Individual (*n* = 186)	11.12 (10.21)	11.35 * (9.14) ^b^	15.39 * (9.20) ^b^	18.93 * (13.13) ^b^
Team (*n* = 191)	9.91 (9.14)	9.09 (7.86)	13.32 (9.16)	16.16 (11.83)
Competitive level				
Recreational (*n* = 105)	12.49 (10.17)	11.40 (9.10)	15.95 (9.16)	19.91 (13.01)
Club (*n* = 126)	9.79 (9.54)	9.33 (7.92)	13.73 (9.34)	16.43 (12.23)
Regional (*n* = 119)	10.09 (9.22)	10.30 (8.67)	14.27 (9.01)	17.33 (12.18)
Elite (*n* = 25)	9.20 (10.39)	9.60 (9.33)	11.76 (9.46)	15.28 (13.40)

Note: ^a^ significantly different from males; ^b^ significantly different from team athletes. * *p* < 0.05; ** *p* < 0.01.

**Table 2:** DASS-21 cut-off scores [6].

	Depression	Anxiety	Stress
Normal	0–9	0–7	0–14
Mild	10–13	8–9	15–18
Moderate	14–20	10–14	19–25
Severe	21–27	15–19	26–33
Extremely Severe	28+	20+	34+

**Table 3:** Model fit indices for cohort 1 (pre-pandemic) and cohort 2 (post-pandemic).

	Criteria [28]	Cohort 1 (N = 427)	Cohort 2 (N = 380)
χ^2^ (df), *p*-value	*p* > 0.05	296.38 (147), *p* < 0.001	394.38 (147), *p* < 0.001
χ^2^/df (cmin/df)	<3	2.02	2.68
RMSEA (90% CI)	≤0.08	0.05 (0.04–0.06)	0.07 (0.059–0.075)
CFI	>0.90	0.95	0.94
TLI	>0.90	0.94	0.92
NFI	>0.90	0.91	0.92

Note: χ ^2^ = chi-square; RMSEA = root mean square error of approximation; CI = confidence interval; CFI = comparative fit index; TLI = Tucker–Lewis index; NFI = normed fit index.

**Table 4:** Distribution of student-athletes across categories of symptom severity pre- to post-pandemic.

	Normal	Mild	Moderate	Severe	Extremely Severe
Depression					
Pre-pandemic	287 (67.2%)	54 (12.6%)	60 (14.1%)	20 (4.7%)	6 (1.4%)
Post-pandemic	203 (53.4%)	44 (11.6%)	75 (19.7%)	33 (8.7%)	25 (6.6%)
Anxiety					
Pre-pandemic	238 (55.7%)	43 (10.1%)	86 (20.1%)	26 (6.1%)	34 (8.0%)
Post-pandemic	180 (47.4%)	27 (7.1%)	65 (17.1%)	34 (8.9%)	74 (19.5%)
Stress					
Pre-pandemic	272 (63.7%)	64 (15%)	54 (12.6%)	32 (7.5%)	5 (1.2%)
Post-pandemic	213 (56.1%)	51 (13.4%)	65 (17.1%)	42 (11.1%)	9 (2.4%)

## Data Availability

Data are available from the first author on reasonable request.

## Supplementary Materials

The following supporting information can be downloaded at: https://www.mdpi.com/article/10.3390/bs14080642/s1, Table S1: Item descriptives for DASS-21; Table S2: Summary of internal reliability for DASS-21 total and sub-scales for cohort 1 and cohort 2; Table S3: Standardised factor loadings and latent factor correlations for bifactor model.
