# Supplementary material for: The Depression, Anxiety, and Stress of Student-Athletes from a Pre- to Post-COVID-19 World
Source: Behav Sci (Basel). 2024 Jul 25;14(8):642. doi: 10.3390/bs14080642 (PMC11352066; doi:10.3390/bs14080642)
Supplement: Supplementary file 1 [file behavsci-14-00642-s001.zip › behavsci-3059876-supplementary.pdf]

Supplementary Table S1. Item descriptives for DASS-21

Item	Cohort 1 (N = 427)				Cohort 2 (N = 380)			
	M	SD	Skewness	Kurtosis	M	SD	Skewness	Kurtosis
1. I found it hard to wind down.	1.34	0.83	0.27	-0.41	1.33	0.89	0.23	-0.65
2. I was aware of dryness of my mouth.	0.90	0.92	0.68	-0.51	0.80	0.84	0.70	-0.47
3. I couldn't seem to experience any positive feeling at all.	0.49	0.62	1.00	0.56	0.70	0.82	1.03	0.44
4. I experienced breathing difficulty (e.g., excessively rapid breathing, breathlessness in the absence of physical exertion).	0.36	0.63	1.72	2.39	0.58	0.82	1.20	0.45
5. I found it difficult to work up the initiative to do things.	1.07	0.78	0.29	-0.43	1.17	0.87	0.34	-0.56
6. I tended to over-react to situations.	0.89	0.81	0.56	-0.35	0.98	0.90	0.52	-0.62
7. I experienced trembling (e.g., in the hands).	0.38	0.66	1.76	2.55	0.58	0.81	1.17	0.35
8. I felt that I was using a lot of nervous energy.	0.77	0.81	0.72	-0.32	1.03	0.94	0.40	-0.93
9. I was worried about situations in which I might panic and make a fool of myself.	0.85	0.88	0.70	-0.48	1.05	0.98	0.47	-0.91
10. I felt that I had nothing to look forward to.	0.47	0.70	1.37	1.24	0.65	0.86	1.14	0.33
11. I found myself getting agitated.	0.91	0.86	0.64	-0.34	1.08	0.93	0.40	-0.82
12. I found it difficult to relax.	1.07	0.87	0.49	-0.43	1.19	0.92	0.30	-0.78
13. I felt down-hearted and blue.	0.73	0.79	0.77	-0.20	0.92	0.89	0.65	-0.43
14. I was intolerant of anything that kept me from getting on with what I was doing.	0.73	0.74	0.73	-0.05	0.75	0.83	0.75	-0.46
15. I felt I was close to panic.	0.47	0.73	1.43	1.24	0.77	0.90	0.90	-0.20
16. I was unable to become enthusiastic about anything.	0.46	0.65	1.31	1.39	0.71	0.85	0.88	-0.31
17. I felt I wasn't worth much as a person.	0.35	0.69	2.16	4.34	0.60	0.90	1.36	0.79
18. I felt I was rather touchy.	0.76	0.78	0.74	-0.07	0.80	0.90	0.89	-0.16
19. I was aware of the action of my heart in the absence of physical exertion (e.g., sense of heart-rate increase, heart missing a beat).	0.54	0.82	1.45	1.28	0.67	0.88	1.07	0.07
20. I felt scared without any good reason.	0.39	0.66	1.56	1.45	0.63	0.83	1.02	-0.09
21. I felt that life was meaningless.	0.23	0.57	2.63	6.89	0.51	0.85	1.59	1.47

Note: Cohort 1 data were collected pre-pandemic (between January 2019 and March 2020) and cohort 2 data were collected post-pandemic (between November 2021 and November 2022).

Supplementary Table S2. Summary of internal reliability for DASS-21 total and sub-scales for cohort 1 and cohort 2

Item	Cohort 1 (N = 427)			Cohort 2 (N = 380)		
	Corrected Item-Total Correlation	Cronbach's Alpha if Item Deleted	Cronbach's Alpha (95% CI)	Corrected Item-Total Correlation	Cronbach's Alpha if Item Deleted	Cronbach's Alpha (95% CI)
**Negative Affectivity (Total)**			0.90 (.89-.92)			0.94 (0.93-0.95)
1. I found it hard to wind down.	0.41	0.90		0.46	0.94	
2. I was aware of dryness of my mouth.	0.25	0.91		0.34	0.94	
3. I couldn't seem to experience any positive feeling at all.	0.56	0.90		0.68	0.94	
4. I experienced breathing difficulty (e.g., excessively rapid breathing, breathlessness in the absence of physical exertion).	0.42	0.90		0.61	0.94	
5. I found it difficult to work up the initiative to do things.	0.48	0.90		0.56	0.94	
6. I tended to over-react to situations.	0.56	0.90		0.62	0.94	
7. I experienced trembling (e.g., in the hands).	0.43	0.90		0.54	0.94	
8. I felt that I was using a lot of nervous energy.	0.61	0.90		0.71	0.94	
9. I was worried about situations in which I might panic and make a fool of myself.	0.60	0.90		0.67	0.94	
10. I felt that I had nothing to look forward to.	0.55	0.90		0.74	0.94	
11. I found myself getting agitated.	0.68	0.90		0.69	0.94	
12. I found it difficult to relax.	0.67	0.90		0.66	0.94	
13. I felt down-hearted and blue.	0.62	0.90		0.74	0.94	
14. I was intolerant of anything that kept me from getting on with what I was doing.	0.44	0.90		0.63	0.94	
15. I felt I was close to panic.	0.63	0.90		0.74	0.94	
16. I was unable to become enthusiastic about anything.	0.63	0.90		0.78	0.94	
17. I felt I wasn't worth much as a person.	0.60	0.90		0.72	0.94	
18. I felt I was rather touchy.	0.55	0.90		0.61	0.94	

19. I was aware of the action of my heart in the absence of physical exertion (e.g., sense of heart-rate increase, heart missing a beat).	0.45	0.90		0.59	0.94	
20. I felt scared without any good reason.	0.58	0.90		0.70	0.94	
21. I felt that life was meaningless.	0.46	0.90		0.66	0.94	
**Depression**			0.85 (0.82-0.87)			0.91 (0.80-0.92)
3. I couldn't seem to experience any positive feeling at all.	0.60	0.83		0.72	0.89	
5. I found it difficult to work up the initiative to do things.	0.50	0.85		0.56	0.91	
10. I felt that I had nothing to look forward to.	0.62	0.83		0.77	0.89	
13. I felt down-hearted and blue.	0.66	0.82		0.75	0.89	
16. I was unable to become enthusiastic about anything.	0.68	0.82		0.81	0.88	
17. I felt I wasn't worth much as a person.	0.65	0.82		0.75	0.89	
21. I felt that life was meaningless.	0.57	0.83		0.71	0.89	
**Anxiety**			0.74 (0.71-0.78)			0.83 (0.80-0.86)
2. I was aware of dryness of my mouth	0.25	0.77		0.32	0.85	
4. I experienced breathing difficulty (e.g., excessively rapid breathing, breathlessness in the absence of physical exertion).	0.47	0.71		0.64	0.80	
7. I experienced trembling (e.g., in the hands).	0.50	0.71		0.58	0.81	
9. I was worried about situations in which I might panic and make a fool of myself.	0.51	0.70		0.59	0.81	
15. I felt I was close to panic.	0.54	0.70		0.71	0.79	
19. I was aware of the action of my heart in the absence of physical exertion (e.g., sense of heart rate increase, heart missing a beat).	0.50	0.70		0.58	0.81	
20. I felt scared without any good reason.	0.53	0.70		0.66	0.80	

**Stress**			0.82 (0.79-0.84)			0.86 (0.83-0.88)
1. I found it hard to wind down.	0.46	0.81		0.51	0.85	
6. I tended to over-react to situations.	0.56	0.79		0.62	0.83	
8. I felt that I was using a lot of nervous energy.	0.53	0.80		0.63	0.83	
11. I found myself getting agitated.	0.65	0.77		0.72	0.82	
12. I found it difficult to relax.	0.70	0.77		0.69	0.82	
14. I was intolerant of anything that kept me from getting on with what I was doing.	0.43	0.81		0.57	0.84	
18. I felt I was rather touchy.	0.55	0.79		0.59	0.84	

Note: CI = confidence interval

Supplementary Table S3. Standardised factor loadings and latent factor correlations for bifactor model.

	Cohort 1 (N = 427)				Cohort 2 (N = 380)			
Item	Total	Depression	Anxiety	Stress	Total	Depression	Anxiety	Stress
**Depression**								
3.	0.468	**0.472**			**0.752**	0.004		
5.	0.374	**0.432**			**0.597**	-0.228		
10.	0.476	**0.499**			**0.821**	-0.166		
13.	0.526	**0.573**			**0.808**	-0.22		
16.	0.523	**0.540**			**0.841**	-0.068		
17.	**0.531**	0.381			**0.761**	0.106		
21.	0.364	**0.410**			**0.731**	0.131		
**Anxiety**								
2.	0.266		**0.307**		**0.322**		0.061	
4.	**0.471**		0.242		**0.579**		0.318	
7.	**0.546**		0.154		**0.499**		0.387	
9.	**0.698**		-0.118		**0.647**		0.189	
15.	**0.718**		-.201		**0.710**		0.375	
19.	**0.510**		0.383		**0.545**		0.423	
20.	**0.628**		-0.062		**0.688**		0.313	
**Stress**								
1.	0.340			**0.454**	0.341			**0.625**
6.	**0.527**			0.270	**0.562**			0.349
8.	**0.722**			-0.021	**0.665**			0.288
11.	**0.674**			0.341	**0.634**			0.386
12.	0.581			**0.650**	0.570			**0.572**
14.	**0.398**			0.247	**0.619**			0.154
18.	**0.519**			0.254	**0.559**			0.237
**Latent Correlations**								
Depression			-0.248	0.448			0.603	-0.490
Anxiety				-0.124				0.484

Note: Values in **bold** indicate highest factor loading.
